# A systematic approach to laparoscopic hysterectomy for enlarged uteri: The Ship Theory

**DOI:** 10.3389/fsurg.2025.1681831

**Published:** 2025-12-02

**Authors:** Stefano Restaino, Giulia Pellecchia, Angelo Finelli, Alessandro Gioè, Martina Arcieri, Stefano Cianci, Federico Paparcura, Alice Poli, Stefano Uccella, Giovanni Scambia, Lorenza Driul, Giuseppe Vizzielli, Salvatore Gueli Alletti

**Affiliations:** 1 Department of Maternal and Child Health, Obstetrics and Gynecology Clinic, University Hospital of Udine, Udine, Italy; 2 PhD School in Biomedical Sciences, Gender Medicine, Child and Women Health, University of Sassari, Sassari, Italy; 3 Department of Medicine, University of Udine, Udine, Italy; 4 Gynecology and Breast Care Center, Mater Olbia Hospital, Olbia, Italy; 5 Ospedale Buccheri La Ferla Fatebenefratelli, Dipartimento Materno-Infantile, UOC Ginecologica e Ostetricia, Palermo, Italy; 6 Unit of Gynecology and Obstetrics, Policlinico “G. Martino”, Department of Human Pathology of Adult and Childhood “G. Barresi”, University of Messina, Messina, Italy; 7 Unit of Gynecology and Obstetrics, Department of Surgery, Dentistry, Pediatrics, and Gynecology, University of Verona, AOUI Verona, Verona, Italy; 8 Department of Woman and Child Health and Public Health, Fondazione Policlinico Universitario A. Gemelli IRCCS, Rome, Italy

**Keywords:** hysterectomy, minimally invasive surgery, fibroid uterus, enlarged uteri, laparoscopy

## Abstract

Total laparoscopic hysterectomy (TLH) for enlarged uteri presents a significant challenge for surgeons due to limited surgical field exposure, increasing the risk of injury to the bowel, bladder, ureters, and blood vessels. To minimize these intraoperative complications, a surgical approach known as “The Ship Theory” has been developed at our center. According to this concept, the uterus is likened to a large vessel moored within the pelvis. As its supporting ligaments (“anchors”) are progressively released, the uterus gains mobility, allowing it to migrate into the abdominal cavity. This enhanced mobility improves visualization and facilitates surgical access, enabling a safer and more effective TLH for large uteri. Using this approach, we successfully performed this procedure on a 51-year-old female patient with uterine leiomyomas and metrorrhagia. Preoperative imaging revealed a uterus measuring 189 × 158 × 148 mm. Institutional review board and ethics committee approval was obtained. The total operative time was approximately 90 min, with an estimated blood loss of less than 50 mL. The patient was discharged 48 h postoperatively without complications. This technical report demonstrates that the application of a minimally invasive surgical technique for uteri with significant spatial constraints—as outlined in “The Ship Theory"—is not only feasible but can be successfully executed when performed in a systematic and structured manner.

## Introduction

The field of minimally invasive gynecologic surgery has made significant advancements over the past three decades. One of the ongoing debates concerns the feasibility and indications for total laparoscopic hysterectomy (TLH) in cases of an enlarged uterus (1, 2). TLH for large uteri presents considerable challenges due to restricted surgical field exposure, increasing the risk of injury to adjacent structures such as the bowel, bladder, ureters, and blood vessels. The latter are often hypertrophied, exhibiting enhanced vascularization and vessel thickening due to increased blood supply and angiogenesis (3). Additionally, laparoscopic surgery has a steep learning curve, which may lead to prolonged operative time and technical difficulties in tissue extraction (4).

A uterus is generally considered large when it extends beyond the confines of the true pelvis, typically corresponding to a weight of approximately 500 g (5). Leiomyomas are the main cause of significant uterine enlargement (6). Training new surgeons to perform and successfully complete such complex procedures remains a significant challenge. To address these difficulties, we have developed a structured procedural framework that enables even surgeons in training to navigate and overcome the technical obstacles associated with large uteri. In this study, supplemented by our instructional video, we aim to demonstrate how adherence to the principles of “The Ship Theory” facilitates the successful execution of a step-by-step TLH for large uteri. Within the framework of The Ship Theory, each metaphorical ‘anchoring point’ is directly mapped to a specific ligamentous or vascular attachment—round ligament, adnexal pedicles, uterine vessels, and uterosacral ligaments—such that the progressive ‘unmooring’ of the uterus conceptually mirrors, step by step, its anatomical detachment and controlled mobilization into the abdominal cavity. This structured approach mitigates the complexity of the procedure, reducing the likelihood of conversion from a minimally invasive approach to open surgery.

## Case presentation and surgical technique

In the context of the “Ship Theory,” the metaphor is used strictly as a pedagogical tool to simplify spatial orientation and procedural sequencing. Each “anchoring point” corresponds to a specific anatomical attachment—the round ligament, utero-ovarian ligament, uterosacral ligament, and vascular pedicles—that must be sequentially released to restore uterine mobility. “Setting sail” represents progressive mobilization of the uterus into the abdominal cavity after detachment from these structures. Thus, while metaphorical in presentation, each conceptual element of the theory directly maps to a reproducible anatomical step.

The patient was a 51-year-old female presenting with metrorrhagia, classified as abnormal uterine bleeding (AUB) and attributed to two uterine leiomyomas. Ultrasonographic evaluation revealed two leiomyomatous nodules measuring 130 × 130 × 120 mm (color score 2/3) and 40 × 40 × 30 mm (color score 1). The overall uterine dimensions were 189 × 158 × 148 mm. The final diagnosis was AUB secondary to leiomyomas, classified according to the FIGO system.

Institutional review board and ethics committee approval was obtained. TLH with bilateral adnexectomy was performed (step-by-step video demonstration attached VC1). Pneumoperitoneum was established using an open laparoscopic access technique with Hadson's trocar at the umbilical/supra-umbilical level or at Palmer's point, particularly in patients with multiple previous vertical midline incisions. Three ancillary trocars were placed along the line between the left anterior superior iliac spine and the supra-umbilical region.

The enlarged uterus was progressively mobilized and detached from its supporting ligaments, following the principles of the “Ship Theory.” According to this concept, the uterus is visualized as a large vessel moored within the pelvis. As its anchoring ligaments are systematically released, the uterus, akin to a ship setting sail, can migrate into the abdominal cavity. This maneuver enhances visualization and facilitates access to the lateral uterine walls, allowing precise identification of the ureter and uterine artery. The uterus was ultimately extracted through a mini-laparotomy after being enclosed in an endobag.

By applying the “Ship Theory” and adhering to a systematic yet straightforward surgical approach, TLH can be performed safely and effectively. The procedure consisted of the following steps:

 1. Opening of the peritoneum at the level of the round ligament and access to the retroperitoneum.
 2. Lateral development of the vesico-uterine space.
 3. Dissection of the pararectal and paravesical spaces.

Early identification and ligation of the uterine arteries at their origin

 4. Identification, coagulation, and bilateral transection of the infundibulopelvic ligament.
 5. Coagulation and transection of the lateral uterine vessels.
 6. Colpotomy and specimen retrieval via endobag-assisted mini-laparotomy.
 7. Colporrhaphy and final hemostasis check.

In our attached video we give a practical demonstration of the surgical technique with explanation (Supplementary Video S1).

Table 1 summarizes the intersection and similarity between The Ship Theory and surgical steps, and even more so between anatomical structures.

**Table 1:** How metaphor and surgical principles have created the ship theory.

Ship theory concept	Anatomical structure/step	Surgical objective
Anchor 1—“Bow mooring”	Round ligament	Entry to retroperitoneum, exposure of pelvic sidewall
Anchor 2—“Lateral tether”	Infundibulopelvic ligament or utero-ovarian ligament	Control of adnexal blood supply
Anchor 3—“Central mooring”	Uterine artery and vein	Early devascularization, reduced blood loss
Anchor 4—“Stern tether”	Uterosacral ligament	Complete mobilization and access to vaginal fornices
Setting sail	Mobilization of uterus into abdominal cavity	Enhanced exposure and access for colpotomy

Just as a ship is freed from its docking points, the uterus must be progressively detached from its ligaments and vascular connections. This begins with peritoneal incision and round ligament transection, followed by systematic dissection of retroperitoneal spaces. With stepwise detachment, the uterus gains mobility and can be positioned strategically, enhancing exposure to lateral pelvic structures. This maneuver prevents excessive traction and reduces the risk of ureteral and vascular injury. Insufflation assists in maintaining a clear operative field, preventing excessive compression of adjacent organs by the enlarged uterus. Once fully mobilized, the uterus can be morcellated or removed via mini-laparotomy in an endobag, depending on size and patient factors.

The operative time was approximately 90 min, with an estimated blood loss of less than 50 mL. Postoperative hematological examinations were within normal limits, with a hemoglobin level of 10 g/dL. Pathological analysis confirmed the diagnosis of leiomyoma. The patient was discharged 48 h after surgery without complications. During follow-up, the patient reported no discomfort.

## Discussion

The “Ship Theory” is a conceptual framework designed to facilitate TLH for enlarged uteri, a procedure traditionally considered technically challenging due to limited surgical field exposure and increased risk of complications. This theory provides a structured, stepwise approach to safely mobilize and extract a large uterus, minimizing the need for conversion to open surgery. The uterus, especially when significantly enlarged, is likened to a large vessel anchored in the pelvis. Similar to how a ship must be methodically freed from its moorings before setting sail, the uterus must be systematically detached from its supporting ligaments and vascular structures before it can be mobilized. Once released, it can “float” into the abdominal cavity, improving visualization and access to critical anatomical structures such as the ureters and uterine arteries.

TLH for an enlarged uterus has consistently posed a challenge for gynecologists due to the inherent spatial constraints, which hinder mobilization and manipulation. These limitations increase the difficulty in identifying anatomical structures, thereby elevating the risk of vascular, intestinal, and ureteral injury.

Uccella et al. has designed Large Uterus Classification System (LUCS) which could predict surgical outcomes and complication rates in women undergoing TLH for large uteri. The LUCS categorizes large uteri into three types based on intraoperative findings of vascular pedicle displacement (uterine and adnexal vessels):

 • Type 1: No significant displacement of uterine or adnexal vascular pedicles [essentially large fundal fibroid(s) without major anatomical shift].
 • Type 2: Cephalad (upward) displacement of the adnexal vascular pedicles, but uterine vessels remain at approximately normal level.
 • Type 3: Displacement of the uterine vessels (with or without adnexal pedicle displacement), often associated with large cervical or lower-segment fibroids; more distorted anatomy.

Authors found type 2 and 3 uteri were independently associated with a greater risk of total complications and concluded this classification could help the surgical team anticipate technical difficulty, guide trocar placement, choice of surgical steps (for example strategies for Type 3 uteri where ureter/uterine vessel displacement is pronounced) and improve planning (7).

The rationale behind our surgical approach lies precisely here in the pre-planning definition on type 2 and 3 uteri by proposing a useful surgical approach to reduce complications risks.

Various other strategies have been proposed to overcome the technical challenges of TLH in large uteri, including the use of mini-laparotomy-assisted TLH, *in situ* morcellation, and robotic assistance.

Compared with these approaches, The Ship Theory emphasizes anatomical dissection and mobility rather than device-dependent solutions. Its primary advantage lies in its structured reproducibility and educational potential for trainees. However, unlike robotic systems, which compensate for limited pelvic space with enhanced dexterity, The Ship Theory depends entirely on systematic dissection and precise identification of landmarks, making anatomical understanding paramount.

The medical literature demonstrates that TLH is feasible even for uteri exceeding 6,000 g, as reported by Siedhoff et al., who currently hold the record for the largest uterus removed laparoscopically (8). However, in routine clinical practice, open abdominal hysterectomy remains the preferred approach worldwide when the uterine fundus reaches the umbilical level (9). Several studies have highlighted that factors such as obesity, prior abdominal surgery, and a large uterus (>500 g) were previously considered absolute contraindications for TLH (10). Twijnstra et al. reported that a uterine weight exceeding 500 g, a BMI >35, and an age >65 years are associated with an increased conversion rate to open surgery (11). Conversely, other researchers have found no correlation between uterine weight and conversion rate (12). Notably, the only factor independently associated with a reduction in complication rates for uteri >1,000 g is the minimally invasive surgical approach (13). An intriguing aspect emerging from the literature on large uteri is the concept of “surgical skills,” which encompasses a combination of im-measurable “environmental” factors related to the surgeon, operating room team, and institutional organization (11). Studies indicate that the only independent predictor of con-version to open surgery is lower surgeon experience (14). In high-volume referral centers, where surgical expertise is continuously refined, experienced surgeons are capable of overcoming technical challenges. Multiple studies confirm the efficacy of the laparoscopic approach for large uteri. However, comparisons among studies are complicated by the frequent reporting of uterine size in gestational weeks rather than weight. Additionally, different cut-off values for relative contraindications to TLH have been suggested, ranging from 250 g to 1,500 g.

Beyond uterine size, one of the primary factors influencing the complexity of TLH for a large uterus is its lateral dimension or the presence of massive subserosal leiomyomas, both of which impede uterine manipulation and limit retroperitoneal access. Our findings support the assertion that uterine weight alone is not a predictor of conversion to open surgery. Instead, multiple authors suggest that the key determinant is the residual intra-abdominal volume (RIAP), which is influenced by factors such as anterior abdominal wall flexibility, ventilation pressure, patient positioning, proper use of uterine manipulators, uterine mobility, surgical expertise, and the preparedness of the operating room (OR) staff (15, 16).

Considering the impact of these factors on the laparoscopic surgeon's visibility and dexterity, several authors advocate performing these procedures in specialized referral centers, where gynecologists, anesthesiologists, and nurses can optimize their technical proficiency (17–19). We propose that the surgical team must achieve complete synchronization of movements and standardize surgical techniques. Once the learning curve for nor-mal-sized uteri is mastered, these skills can be effectively adapted for larger uteri (20).

The technique described is easily reproducible, but in our report it corresponds to a single application. In any case, before describing the technique, the team of surgeons applied and tested it in various situations, establishing its effectiveness.

## Conclusions

The application of minimally invasive surgical techniques for uteri with significant spatial constraints is not an impossible task. When performed systematically, following and completing a series of simple and precise surgical micro-steps, this approach can be successfully implemented. The Ship Theory may offer a structured, reproducible method to overcome technical challenges in TLH for large uteri. It enhances surgical efficiency, reduces blood loss, and minimizes conversion rates.

This report however, actually represents just a proof of concept demonstrating that the “Ship Theory” framework can guide a safe and reproducible approach to TLH in cases of markedly enlarged uteri. While the findings are encouraging, they are limited to a single clinical case. Further validation through larger prospective studies is necessary to assess reproducibility, learning curve impact, and comparative safety vs. conventional approaches.

Future perspectives would be to validate this proof-of-concept on larger cohorts, including proposed outcome measures (conversion rate, operative time, complication rate) allowing to eventually standardize this surgical procedure.

In conclusion, by implementing The Ship Theory and demonstrating its generalisable applicability on a large sample, surgeons could navigate the complexities of large uterus hysterectomy in a systematic, controlled, and minimally invasive manner, much like a ship being set free from its moorings and guided safely out to sea.

## Data Availability

The original contributions presented in the study are included in the article/Supplementary Material, further inquiries can be directed to the corresponding author.

## Supplementary material

Supplementary Video S1 The accompanying instructional video (VC1) illustrates the key operative stages of The Ship Theory technique. It highlights trocar placement, stepwise dissection of retroperitoneal spaces, early identification of uterine arteries, and visualization of the uterus “floating” into the abdominal cavity after sequential detachment. The video also demonstrates ergonomic positioning, uterine manipulation techniques, and safety checks, serving as a visual complement for surgical education.
